# Mn(II)-Activated Zero-Dimensional Zinc(II)-Based Metal Halide Hybrids with Near-Unity Photoluminescence Quantum Yield

**DOI:** 10.3390/ma17030562

**Published:** 2024-01-25

**Authors:** Chengyu Peng, Jiazheng Wei, Lian Duan, Ye Tian, Qilin Wei

**Affiliations:** 1 Traffic Information Engineering Institute, Guangxi Transport Vocational and Technical College, Nanning 530004, China; 2 School of Semiconductors and Physics, North University of China, Taiyuan 030051, China; 3 School of Chemistry and Chemical Engineering, Shandong University, Jinan 250100, China

**Keywords:** low-dimensional metal halide, lead free, low toxicity, high stability, (TDMP)ZnBr_4_

## Abstract

As derivatives of metal halide perovskite materials, low-dimensional metal halide materials have become important materials that have attracted much attention in recent years. As one branch, zinc-based metal halides have the potential for practical applications due to their lead-free, low-toxicity and high-stability characteristics. However, pure zinc-based metal halide materials are still limited by their poor optical properties and cannot achieve large-scale practical applications. Therefore, in this work, we report an organic–inorganic hybrid zero-dimensional zinc bromide, (TDMP)ZnBr_4_, using transition metal Mn^2+^ ions as dopants and incorporating them into the (TDMP)ZnBr_4_ lattice. The original non-emissive (TDMP)ZnBr_4_ exhibits bright green emission under the excitation of external UV light after the introduction of Mn^2+^ ions with a PL peak position located at 538 nm and a PLQY of up to 91.2%. Through the characterization of relevant photophysical properties and the results of theoretical calculations, we confirm that this green emission in Mn^2+^:(TDMP)ZnBr_4_ originates from the ^4^T_1_ → ^6^A_1_ optical transition process of Mn^2+^ ions in the lattice structure, and the near-unity PLQY benefits from highly localized electrons generated by the unique zero-dimensional structure of the host material (TDMP)ZnBr_4_. This work provides theoretical guidance and reference for expanding the family of zinc-based metal halide materials and improving and controlling their optical properties through ion doping.

## 1. Introduction

Lead-based metal halide perovskites with the general structural formula ABX_3_ have received widespread attention from the scientific community in recent years due to their excellent optoelectronic properties, and they have been used in solar cells, light-emitting diodes, photodetectors and other fields [1,2,3,4,5,6,7,8,9,10,11,12,13,14]. Although remarkable achievements have been made in the field of optoelectronic applications, the inherent toxicity and instability of lead-based materials limit their further practical applications [15,16]. How to improve the stability of materials and reduce the environmental toxicity of Pb^2+^ ions while maintaining excellent optoelectronic properties has become an urgent problem to be solved.

Low-dimensional metal halide materials developed based on ABX_3_ metal halide perovskites are ideal materials to replace lead-based metal halide materials [17,18]. In recent years, low-dimensional metal halide luminescent materials have been widely reported; in particular, Sb(III)-based, In(III)-based and Mn(II)-based materials have become typical representatives due to their high-efficiency luminescent properties and low toxicity [19,20,21,22,23,24]. In addition, low-dimensional Zn(II)-based materials based on Zn with a d^10^ electron configuration have gradually attracted the attention of researchers in the field in recent years due to their variable crystal structure and low-toxicity properties [25,26]. McWhorter et al. reported a Zn-based zero-dimensional metal halide, (C_7_H_11_N_2_)_2_ZnBr_4_, with white light emission with a PLQY of up to 7.35% [25]. At the same time, they also used single crystals of the compound to prepare X-ray detectors and explored future practical applications of Zn-based low-dimensional halide materials. Furthermore, they reported another Zn-based organic–inorganic hybrid metal halide, (P-xd) ZnCl_4_, with white light emission. This compound reached the current maximum white light emission PLQY in Zn-based materials of 23.06% [27]. In addition to broadband white light emission, Zn-based low-dimensional halide materials can also produce other colors of light. For example, R_2_ZnCl_4_ (R = (E)-4-styrylpyridinium, C_13_H_12_N^+^) with bright green emission, [N-EPD]_2_ZnBr_4_ with broadband yellow emission and (Ph_3_S)_2_ZnCl_4_ with blue emission have been reported [28,29,30]. Unfortunately, the optical properties of these materials are poor, which is not conducive to future practical applications. In addition to single-mode light emission, some Zn-based materials also have the characteristic of tunable light emission. Ma et al. reported a kind of (BPP)ZnX_2_ (X = Cl, Br) with a tunable emission color and ultralong room-temperature phosphorescence. Combining the results of experiments and theoretical calculations, they found that the dynamic polychromatic afterglow at room temperature can be attributed to the emission of the ligand BPP and its interaction with halogen atoms [31]. In addition, Wei et al. also reported a BAPPZn_2 × 8_ material with ultralong room-temperature phosphorescence, whose luminescence color is adjustable with the excitation wavelength and applied to the field of optical anti-counterfeiting [32]. Li et al. used a large-sized cyclic organic molecule, 2,5-bis(4-pyridinium)thiazolo [5,4-d]thiazole, as the A-site organic ligand and combined it with zinc halide to prepare a zero-dimensional structure of (H_2_TTz)ZnX_4_·MeOH (X = Cl, Br); the emission wavelength could switch from 462 nm to 512 nm, and they used Br to replace Cl in the lattice structure. Further, they explored the application of these two materials in the field of optical waveguides and found that both compounds had excellent optical waveguide properties. The optical loss coefficient R of (H_2_TTz)ZnCl_4_·MeOH at 462 nm was 0.01577 dB·μm^−1^, and that of (H_2_TTz)·ZnBr_4_·MeOH at 515 nm was 0.01182 dB·μm^−1^ [33]. These results revealed the great potential of low-dimensional Zn-based metal halide materials in photonic technology and provided new inspiration for the practical application of low-loss optical waveguides.

In summary, not only have many Zn-based halide materials been designed, developed and reported, but also a preliminary exploration of future practical applications has been carried out. However, the optical properties of the pure Zn-based metal halides reported so far still have inherent flaws and need to be further improved. How to improve the optical properties of Zn-based materials is of great significance to promoting their future commercial applications. Ion doping is an effective strategy to improve the optical properties of materials [34,35]. By introducing specific optically active ions into a non-luminescent matrix material, efficient luminescence can be achieved without changing the crystal structure of the matrix material. Taking the most widely reported and most intensively studied all-inorganic Cs-Zn-X (Cs_2_ZnX_4_ and Cs_3_ZnX_5_, X = Cl, Br, I) system as an example, many optically active ions have been doped into Cs-Zn-X and achieved a variety of efficient luminescent colors. The incorporation of Cu^+^ ions into Cs-Zn-X can achieve violet-to-blue light emission [36,37,38]. Cu^+^ ions usually exhibit blue emission after doping in chloride and bromide, while in iodide, they exhibit violet emission. Divalent Mn^2+^ ions usually form tetrahedrons in Zn-based halide systems and exhibit green light emission, and Sn^2+^ or Sb^3+^ exhibit deep red to near-infrared emission [39,40,41,42]. In addition to single doping, Cs-Zn-X co-doped with different luminescent ions has also received widespread attention. Wang et al. reported the tunable emission of Mn^2+^/Cu^+^ co-doped Cs_2_ZnBr_4_ and explored the application in the field of optical anti-counterfeiting [43]. Guo et al. reported Sb^3+^/Mn^2+^ co-doped Cs_2_ZnCl_4_ with dual-band emission. This material showed different luminescence responses at different temperatures. They built tunable thermochromic luminescent materials that can be used in applications such as thermal sensing and optical encryption [44]. Zeng et al. also reported Pb^2+^/Mn^2+^ co-doped Cs_2_ZnBr_4_ microcrystals with single-matrix white light emission. White light comes from the cooperative emission of Pb^2+^ ions and Mn^3+^ ions. At the same time, this white light emission can also be tuned from cold white to warm white by controlling the doping concentration of Pb^2+^ ions [45].

Inspired by ion doping, we used the protonated large-size cyclic organic molecule trans-2,5-dimethylpiperazine (C_6_H_14_N_2_, TDMP) as the A-site cation and combined it with zinc bromide to prepare a zero-dimensional organic–inorganic hybrid zinc bromide, (TDMP)ZnBr_4_. Optically active Mn^2+^ ions were introduced into the crystal lattice of non-luminescent (TDMP)ZnBr_4_ as dopants, and bright green light emission (λ_em_ = 538 nm) with a PLQY of up to 91.2% under UV light excitation was achieved. Through optical property characterization and first-principles calculations, it was confirmed that the bright green emission in Mn^2+^:(TDMP)ZnBr_4_ comes from the d-d transition of Mn^2+^ ions, and near-unity PLQY is related to the zero-dimensional structure of (TDMP)ZnBr_4_. The zero-dimensional structure is conducive to the generation of a strong quantum confinement effect and dielectric confinement effect, which can have a strong binding effect on photogenerated excitons and ensure an efficient radiation recombination process, thereby obtaining an ultra-high PLQY. This work provides a certain reference for improving the optical properties of Zn-based metal halide materials and lays a theoretical foundation for their future commercial applications.

## 2. Results and Discussion

### 2.1. Structure and Morphology

**Figure 1:**
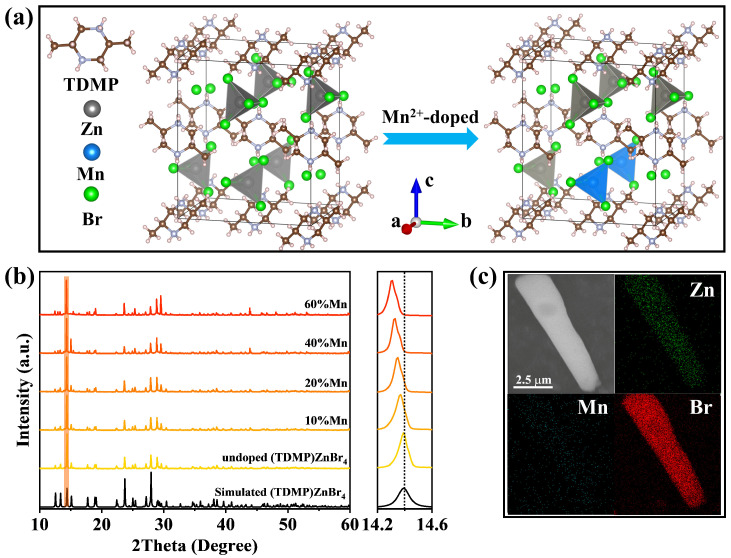
(**a**) Crystal structure of (TDMP)ZnBr_4_ and Mn^2+^:(TDMP)ZnBr_4_. (**b**) XRD patterns of (TDMP)ZnBr_4_ with different Mn^2+^ ion feed ratios. (**c**) SEM image and element mapping of Mn^2+^:(TDMP)ZnBr_4_.

The crystal structure of the host material (TDMP)ZnBr_4_ is shown on the left side of Figure 1a. The (TDMP)ZnBr_4_ crystalline in the C2/c space group is as previously reported [46]. A (TDMP)ZnBr_4_ unit cell consists of four [ZnBr_4_]^2−^ tetrahedrons and fifteen protonated TDMP molecules. [ZnBr_4_]^2−^ tetrahedrons are periodically arranged in the structural skeleton composed of protonated TDMP molecules. Adjacent [ZnBr_4_]^2−^ tetrahedrons are fully separated by protonated TDMP molecules and exhibit isolated distribution at the molecular level. Therefore, there are no interactions between adjacent [ZnBr_4_]^2−^ tetrahedrons, which is typical of zero-dimensional structures. There are two types of Zn-Br bonds in each [ZnBr_4_]^2−^ tetrahedron, with bond lengths of 2.3964 Å and 2.4248 Å. These two bond lengths are very similar, indicating that the structural distortion of the [ZnBr_4_]^2−^ tetrahedron is very small. It is worth noting that, when Mn^2+^ ions are incorporated into the (TDMP)ZnBr_4_ lattice, they replace the lattice positions of Zn^2+^ ions to form [MnBr_4_]^2−^ tetrahedrons, which indicates that the doping of Mn^2+^ ions does not change the crystal structure of (TDMP)ZnBr_4_. As shown on the right side of Figure 1a, a unit cell of Mn^2+^:(TDMP)ZnBr_4_ contains the metal–halogen tetrahedron, with Zn and Mn occupying the site of the tetrahedron center and protonated TDMP molecule. In Mn^2+^:(TDMP)ZnBr_4_, the metal–Br bond length of the tetrahedron is the same as the undoped system. There are two types of metal–Br bonds, with bond lengths of 2.5375 Å and 2.5675 Å. The deformation of tetrahedrons is also very small in Mn^2+^:(TDMP)ZnBr_4_, which indicates that the doping of Mn^2+^ ions does not cause local lattice distortion of the host material. In addition, the bond length of the metal–Br bond becomes larger after Mn^2+^ doping, indicating that the doping of Mn^2+^ ions causes the expansion of the tetrahedron. This is due to the different ionic radii of Mn^2+^ and Zn^2+^. In the case of four coordination, the ionic radius of Mn^2+^ is 0.66 Å, and the ionic radius of Zn^2+^ is 0.60 Å [47]. The incorporation of larger Mn^2+^ ions into the crystal structure will inevitably lead to the expansion of the lattice, which will increase the bond length of the metal–Br in the tetrahedron. The XRD patterns of (TDMP)ZnBr_4_ samples with different Mn^2+^ ion feed ratios are shown in Figure 1b. The undoped (TDMP)ZnBr_4_ lattice structure files used for simulation calculations were obtained from the Cambridge Crystallographic Data Center (CCDC-1424478). The XRD patterns of all (TDMP)ZnBr_4_ samples are in good agreement with those calculated using simulations, and no redundant diffraction peaks appear, indicating that Mn^2+^:(TDMP)ZnBr_4_ were successfully synthesized and that the samples were pure and free of impurities. At the same time, the diffraction peak of the sample moves to a low angle direction as the Mn^2+^ ion feed ratio increases. This result indicates the successful doping of Mn^2+^ ions, because the incorporation of Mn^2+^ ions with a larger radius will cause the lattice to expand and shift the diffraction peak to a lower angle direction. The surface morphology of Mn^2+^:(TDMP)ZnBr_4_ microcrystals and their distribution of elements were observed using a scanning electron microscope (SEM) and energy-dispersive X-ray spectrometer attachment (EDS), which obtain by HITACHI SU8020 (HITACHI, Tokyo, Japen). Figure 1c shows the surface morphology of the Mn^2+^:(TDMP)ZnBr_4_ microcrystals and their corresponding element distribution. The surface of rod-shaped Mn^2+^:(TDMP)ZnBr_4_ microcrystals is smooth, indicating that the microcrystalline samples synthesized through solvothermal synthesis have good crystallinity and high crystal quality. The results of the elemental analysis show that Zn, Mn and Br elements are evenly distributed in the crystal, and the atomic ratio of the three elements is Zn:Mn:Br = 20.4:0.95:78.65 = 1:0.047:3.855 (Figure S1). The atomic ratio of Zn to Br is close to the stoichiometric ratio of 1:4 in (TDMP)ZnBr_4_, which further demonstrates the successful synthesis of (TDMP)ZnBr_4_ and the doping of Mn^2+^ ions. In addition, the Raman spectrum of undoped (TDMP)ZnBr_4_ and Mn^2+^:(TDMP)ZnBr_4_ obtained with a 633 nm continuous laser as the excitation source was also collected, as shown in Figure S2. Figure S2a shows the inorganic part of the Raman spectra. The Raman mode in the low wavenumber (<400 cm^−1^) range originates from the vibrations of inorganic [ZnBr_4_]^2−^ and [MnBr_4_]^2−^ tetrahedrons [48]. The Raman mode of the Mn^2+^ ion-doped sample shifts to a low wavenumber direction compared with the undoped sample, which is consistent with the XRD pattern. This result also proves that Mn^2+^ ions were successfully doped in the structure of (TDMP)ZnBr_4_, because the expansion or contraction of the crystal lattice causes a shift in the Raman peak [49]. The Raman spectrum of the organic part demonstrates that the protonated organic TDMP molecules were successfully inserted into the lattice of inorganic ZnBr_2_ (Figure S2b).

### 2.2. Optical Properties

**Figure 2:**
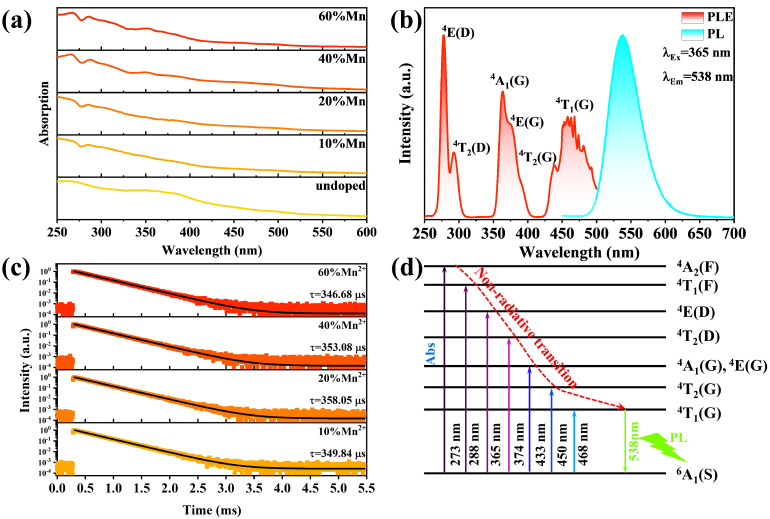
(**a**) The absorption spectrum of (TDMP)ZnBr_4_ under different Mn^2+^ ion feed ratios. (**b**) The PLE spectra and PL spectra of Mn^2+^:(TDMP)ZnBr_4_. (**c**) The PL decay lifetime curve of (TDMP)ZnBr_4_ under different Mn^2+^ ion feed ratios. (**d**) Schematic diagram of the photophysical mechanism of Mn^2+^:(TDMP)ZnBr_4_.

The optical properties of Mn^2+^:(TDMP)ZnBr_4_ were characterized to explore their underlying photophysical mechanisms. First, a Lambda-750 UV-vis-NIR spectrophotometer was used to collect the absorption spectra of (TDMP)ZnBr_4_ with different Mn^2+^ ion feed ratios. As shown in Figure 2a, undoped (TDMP)ZnBr_4_ absorbs in the range of 250 nm to 550 nm, and the absorption intensity gradually increases as the wavelength decreases. Three new absorption bands appear at 267 nm, 285 nm and 353 nm when Mn ions are incorporated, and the intensity of these three absorption bands increases with the increase in the Mn^2+^ ion feed ratio. Obviously, these absorption bands come from the d-d transition absorption of Mn^2+^ ions. According to the elemental analysis results, the actual Mn^2+^ ions incorporated into the (TDMP)ZnBr_4_ lattice are still quite limited, even though the feed ratio of Mn^2+^ ions reaches 40% or even 60%. The following formula was used to calculate the bandgap value of (TDMP)ZnBr_4_ with different Mn^2+^ ion feed ratios [50]:

[*F*(*R*_∞_)*hυ*]*^n^* = *A*(*hυ* − *E_g_*)

where *F*(*R*_∞_) is the Kubelka–Munk (K-M) equation, *hυ* is the photon energy, *A* is the constant, and *E_g_* is the bandgap. The value of *n* depends on the bandgap properties of the semiconductor. *n* = 2 for the direct bandgap semiconductor, and *n* = 1/2 for the indirect bandgap semiconductor. Since (TDMP)ZnBr_4_ is a direct bandgap semiconductor, the bandgap values of the (TDMP)ZnBr_4_ samples with different Mn^2+^ ion feed ratios were calculated according to the above formula, and the results are shown in Figure S3. The bandgap of the undoped (TDMP)ZnBr_4_ sample is 3.71 eV, and the bandgap will slightly reduce while Mn^2+^ ions are introduced.

The photoluminescence excitation spectrum (PLE) and photoluminescence (PL) spectrum of Mn^2+^:(TDMP)ZnBr_4_ are shown in Figure 2b. Mn^2+^:(TDMP)ZnBr_4_ exhibits bright green emission under the excitation of 365 nm UV light, with the PL peak located at 538 nm; the full width of half maximum (FWHM) is 51.71 nm, and the Stokes shift is 173 nm. It is worth noting that the green emission of Mn^2+^:(TDMP)ZnBr_4_ is not completely standard green. The CIE coordinate of this green emission is (0.30, 0.67) and located at the edge of the green light area, as shown in Figure S4. The PLE spectrum of Mn^2+^:(TDMP)ZnBr_4_ was collected at the PL position of 538 nm. Obvious excitation bands can be observed at 275 nm, 291 nm, 360 nm, 375 nm, 388 nm and 462 nm, which belong to the absorption transitions of ^6^A_1_ to ^4^E(D), ^4^T_2_(D), ^4^A_1_(G), ^4^E(G), ^4^T_2_(G) and ^4^T_1_(D) of Mn^2+^ ions, respectively [51,52]. The excitation band in the PLE spectrum does not observe the contribution of the host material (TDMP)ZnBr_4_ to the 538 nm green emission. All the excitation bands come from the absorption transition of Mn^2+^ ions, which shows that the optical transition process of Mn^2+^:(TDMP)ZnBr_4_ is dominated by the Mn^2+^ ions doped in the crystal lattice. The PL spectra of (TDMP)ZnBr_4_ with different Mn^2+^ ion feed ratios are shown in Figure S5. As the feed ratio increases, the PL intensity of (TDMP)ZnBr_4_ first increases and reaches the maximum when the Mn^2+^ feed ratio is up to 20%. Then, the PL intensity of (TDMP)ZnBr_4_ decreases as the feed ratio of Mn^2+^ ions is further increased, which is caused by the concentration quenching effect under high-concentration doping. The PLQY of the Mn^2+^-doped sample was collected using 365 nm UV light as the excitation source. The measurement results show that Mn^2+^:(TDMP)ZnBr_4_ reaches the maximum of 91.2% when the Mn^2+^ ion feed ratio is 20%, as shown in Figure S6. In order to further explore the origin of green emission in Mn^2+^:(TDMP)ZnBr_4_, we collected the PL spectra under different excitation wavelengths and the PLE spectra under different emission wavelengths, as shown in Figure S7. The PLE spectra of Mn^2+^:(TDMP)ZnBr_4_ collected at different emission wavelengths were normalized. We found that the obtained PLE spectra all have the same profile as the emission wavelength increases from 520 nm to 550 nm, as shown in Figure S7a. At the same time, the PL spectra obtained at different excitation wavelengths also show the same profile (Figure S7b). This indicates that the 538 nm green emission in Mn^2+^:(TDMP)ZnBr_4_ originates from the same excited state.

The PL lifetime decay curves of (TDMP)ZnBr_4_ under different Mn^2+^ ion feed ratios were collected to confirm the source of green emission. As shown in Figure 2c, all Mn^2+^:(TDMP)ZnBr_4_ samples exhibit hundreds of microseconds PL decay lifetimes. This long lifetime of hundreds of microseconds to tens of milliseconds is a typical feature of the Mn^2+^ ions’ d-d transition (^4^T_1_ → ^6^A_1_) [51,53,54]. Combining the PLE spectrum and PL spectrum results, we confirmed that the 538 nm green emission in Mn^2+^:(TDMP)ZnBr_4_ originates from the ^4^T_1_ → ^6^A_1_ radiation transition of Mn^2+^ ions. Therefore, the photophysical processes in Mn^2+^:(TDMP)ZnBr_4_ can be summarized as follows: Optically active Mn^2+^ ions are introduced into non-luminescent (TDMP)ZnBr_4_. Mn^2+^ ions replace the lattice site of Zn^2+^ ions and combine with the surrounding four Br^−^ ions to form [MnBr_4_]^2−^ tetrahedrons when they enter the (TDMP)ZnBr_4_ lattice. According to the crystal field theory, the crystal field intensity experienced by the four-coordinated Mn^2+^ ions is weak. The energy level difference between the ^4^T_1_ and ^6^A_1_ energy levels is large, leading to high-energy green emission under the excitation of UV light. According to the results of the PLE spectrum, the host material does not contribute to the emission; hence, the excitation–emission process in Mn^2+^:(TDMP)ZnBr_4_ occurs entirely at the d energy level of the Mn^2+^ ions. As shown in Figure 2d, under UV light excitation, electrons at the ground-state energy level ^6^A_1_ absorb the energy of UV light and transition to excited states of higher energy levels. Subsequently, the electrons in the high-energy excited state will return to the lowest energy level ^4^T_1_ excited state through non-radiative transition and, finally, transition back to the ground state through radiative recombination producing 538 nm green emission.

**Figure 3:**
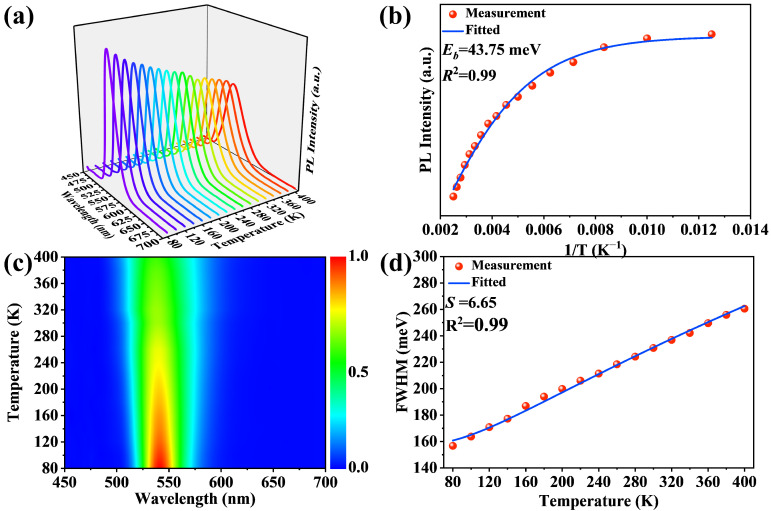
(**a**) The temperature-dependent PL spectra of Mn^2+^:(TDMP)ZnBr_4_. (**b**) The activation energy fitting results of Mn^2+^:(TDMP)ZnBr_4_. (**c**) The pseudo-color image of the Mn^2+^:(TDMP)ZnBr_4_ temperature-dependent PL spectrum. (**d**) The fitting result of FWHM and temperature.

In order to deeply study the relationship between the crystal structure and photophysical properties, PL spectra of Mn^2+^:(TDMP)ZnBr_4_ in the temperature range of 80–400 K were collected. As shown in Figure 3a, Mn^2+^:(TDMP)ZnBr_4_ exhibits single-band emission in the entire temperature range from 80 K to 400 K, without additional emission bands appearing with temperature changes. This indicates that Mn^2+^:(TDMP)ZnBr_4_ has a high structural stability and that the crystal structure will not undergo phase changes with drastic changes in temperature, because phase changes may generate new emission bands. The PL intensity at each temperature was extracted from the temperature-dependent PL spectrum and used as the vertical axis with the reciprocal of temperature as the horizontal axis, and a scatter plot of the changing trend of PL intensity with the reciprocal of temperature was drawn. The scatter plot was fitted using the Arrhenius formula to obtain the activation energy *E_b_* of Mn^2+^:(TDMP)ZnBr_4_ [52]:

$$\;I\left(T\right)=\frac{I_{0}}{\left(1+Ae^{\frac{-E_{b}}{k_{b}T}}\right)}$$

where *I*_0_ is the PL intensity at 0 K, *I*(*T*) is the PL intensity at temperature K, and *k_b_* is the Boltzmann constant. The activation energy *E_b_* obtained by fitting is 43.75 meV, which is much higher than the room-temperature thermal energy (~26 meV) [55]. This indicates that the emission of Mn^2+^:(TDMP)ZnBr_4_ has strong resistance to thermal quenching. A larger activation energy means that the dissociation process requires more energy, which also indicates that the excitons are more stable and have stronger resistance to thermal quenching [56]. This means that, when the temperature reaches 400 K, Mn^2+^:(TDMP)ZnBr_4_ can still maintain a certain intensity of emission, as shown in the pseudo-color image of the temperature-dependent PL spectrum (Figure 3c). In addition, we also extracted the FWHM of the Mn^2+^:(TDMP)ZnBr_4_ spectrum at each temperature and fitted the FWHM–temperature change curve using the following formula [57]:

$$\mathrm{FWHM}\left(T\right)=2.36\sqrt{S}\hbar \omega_{\mathrm{phonon}}\sqrt{\coth\frac{\hbar \omega_{\mathrm{phonon}}}{2k_{b}T}}$$

where *S* is the Huang–Rhys factor, *ω*_phonon_ is the phonon frequency, *T* is the temperature, FWHM is the full-width half maximum of the PL spectra, and *k_b_* is the Boltzmann constant. The fitting results are shown in Figure 3d, and Huang–Rhys factor *S* obtained by fitting is 6.65. Such a small *S* value indicates that the emission of Mn^2+^:(TDMP)ZnBr_4_ comes from the d-d transition of Mn^2+^ ions rather than self-trapped exciton (STE) emission caused by local lattice distortion because STE emission requires a certain intensity of the electron–phonon coupling effect, and the *S* value obtained by fitting is larger [58]. In addition, the measurement results of the Raman spectrum also confirm this result. Strong Raman scattering at room temperature is an important indicator of strong coupling between excited-state electrons and lattice vibrations [59]. Therefore, strong scattering interactions reflect the tendency of local fluctuations in the lattice. This propensity to form localized charges plays a role in charge transport, as free charge carriers cause lattice deformation, which moves with the carriers and forms self-trapped excitons. The Raman spectrum in Figure S2 shows a very weak Raman scattering intensity, which indicates that the coupling between its electrons and lattice vibration is too weak to produce STE emission. At the same time, cyclic organic molecules with greater structural rigidity are also an important reason for the smaller *S* value, because the lattice vibration produced by rigid organic molecules is smaller [60].

### 2.3. Theoretical Calculation

**Figure 4:**
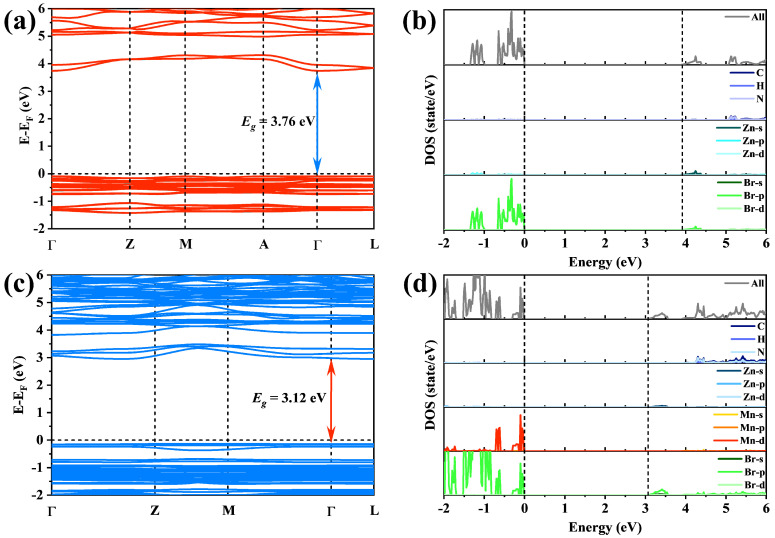
(**a**) The bandgap structure of (TDMP)ZnBr_4_. (**b**) The DOS of (TDMP)ZnBr_4_. (**c**) The bandgap structure of Mn^2+^:(TDMP)ZnBr_4_. (**d**) The DOS of Mn^2+^:(TDMP)ZnBr_4_.

In order to study the relationship between the Mn^2+^:(TDMP)ZnBr_4_ electronic structure and luminescence properties, we performed first-principles calculations on (TDMP)ZnBr_4_ before and after Mn^2+^ ion doping. Figure 4a,c present the energy band structure of (TDMP)ZnBr_4_ before and after Mn^2+^ ion doping, respectively. The pristine (TDMP)ZnBr_4_ bandgap value obtained by first-principles calculations is 3.76 eV, while the bandgap value of Mn^2+^:(TDMP)ZnBr_4_ is 3.12 eV. The calculation results show that the doping of Mn^2+^ ions shrinks the bandgap of the host material, which is consistent with the actual bandgap change in the samples obtained using the tauc plot method. Figure 4b,d show the density of state (DOS) of (TDMP)ZnBr_4_ and Mn^2+^:(TDMP)ZnBr_4_, respectively. In undoped (TDMP)ZnBr_4_, the conduction band minimum (CBM) is formed by Br-4p orbitals and Zn-5s orbitals, while the valence band maximum (VBM) is mainly formed by Br-5p orbitals, as shown in Figure 4b. The VBM of Mn^2+^:(TDMP)ZnBr_4_ is formed by Br-4p orbitals and Mn-3d orbitals, while the CBM is mainly formed by Br-4p orbitals, as shown in Figure 4d. The theoretical calculation results indicate that the Zn-5s orbital involved in band-edge formation is replaced by the Mn-3d and Br-4p orbitals when Mn^2+^ ions are incorporated. This indicates that [MnBr_4_]^2−^ tetrahedrons dominate the main optical transition process in Mn^2+^:(TDMP)ZnBr_4_ after doping. The UV light directly excites Mn^2+^ ions in the lattice structure, and all optical processes occur between the d orbital energy levels of Mn^2+^ ions. This also explains why the contribution of the host material to 538 nm green emission cannot be observed in the PLE spectrum. Organic cations do not contribute to the formation of frontier orbits but could affect the optical processes by producing spatial confinement effects and dielectric confinement effects on [MnBr_4_]^2−^ tetrahedrons. At the same time, Mn^2+^:(TDMP)ZnBr_4_ shows a relatively flat conduction band and valence band with very small dispersion, which indicates that the electrons are highly localized (Figure 4c). This band structure is unique to the 0D structure [22]. Each [MnBr_4_]^2−^ tetrahedron is separated by organic cations to form the highly localized electrons in Mn^2+^:(TDMP)ZnBr_4_, thus generating the isolated luminescent centers. This highly localized electron is a key factor in producing efficient luminescence [22,61].

### 2.4. Stability

**Figure 5:**
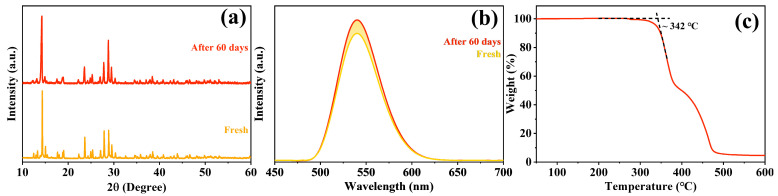
(**a**) The XRD patterns of Mn^2+^:(TDMP)ZnBr_4_ before and after 60 days. (**b**) The PL spectra of Mn^2+^:(TDMP)ZnBr_4_ before and after 60 days. (**c**) The thermal decomposition curve of Mn^2+^:(TDMP)ZnBr_4_.

The stability of a material is an important indicator of whether it has practical application value. Here, we determined the structure, luminescence and thermal stability of Mn^2+^:(TDMP)ZnBr_4_ to evaluate its potential for practical applications in the future. As shown in Figure 5a, the XRD diffraction pattern of Mn^2+^:(TDMP)ZnBr_4_ after being placed under ambient conditions for 60 days is basically consistent with the diffraction pattern of the as-synthesized sample, and no additional impurity diffraction peaks were observed, indicating that this compound can retain a stable structure without decomposition for a long time. Meanwhile, Mn^2+^:(TDMP)ZnBr_4_ also has high PL stability. The PL intensity of Mn^2+^:(TDMP)ZnBr_4_ does not decrease significantly and remains at 90–95% of the initial PL intensity after being placed under ambient conditions for 60 days (Figure 5b), and it can exhibit bright green emission under UV light excitation. Finally, the results of the thermogravimetric analysis show that Mn^2+^:(TDMP)ZnBr_4_ also has high thermal stability. As shown in Figure 5c, the decomposition temperature of Mn^2+^:(TDMP)ZnBr_4_ is about 342 °C. All experiments, including synthesis, characterization and storage, were performed under ambient conditions, with a relative humidity of 50–70% without any inert gas protection. The stability test results show that Mn^2+^:(TDMP)ZnBr_4_ has great structural, luminescence and thermal stability, and it has application potential in the field of future optoelectronic devices.

## 3. Conclusions

In summary, we selected transition metal Mn^2+^ ions as sensitizers and introduced them into a non-luminescent (TDMP)ZnBr_4_ lattice in this work. Under the excitation of 365 nm UV light, Mn^2+^:(TDMP)ZnBr_4_ exhibits bright green light emission (λ_em_ = 538 nm) with a PLQY of up to 91.2%. Combining optical property characterization and first-principles calculations, we confirmed that the 538 nm green light emission in Mn^2+^:(TDMP)ZnBr_4_ originates from the ^4^T_1_ → ^6^A_1_ transition of Mn^2+^ ions in the lattice structure. The unique 0D structure of the host material can generate a strong quantum confinement effect and dielectric confinement effect, which is very beneficial to the generation of highly localized excitons. The photogenerated excitons will be bound within the tetrahedron by the strong confinement effect of the crystal lattice once they are formed by outside excitation, and they will radiate photons outward through radiative recombination, ultimately producing efficient green light emission. Otherwise, Mn^2+^:(TDMP)ZnBr_4_ also has high structural, luminescence and thermal stability, which further enhances the competitiveness of this compound in future practical applications. This work provides theoretical guidance and references for expanding the zinc-based metal halide family and improving its optical properties via ion doping.

## Data Availability

Data are contained within the article.

## Supplementary Materials

The following supporting information can be downloaded at https://www.mdpi.com/article/10.3390/ma17030562/s1, Figure S1: EDS spectra, Figure S2: Raman spectra, Figure S3: Bandgap value, Figure S4: CIE coordination, Figure S5: PL spectra, Figure S6: PLQY, Figure S7: PLE and PL spectra.
