# Supplementary material for: Mn(II)-Activated Zero-Dimensional Zinc(II)-Based Metal Halide Hybrids with Near-Unity Photoluminescence Quantum Yield
Source: Materials (Basel). 2024 Jan 25;17(3):562. doi: 10.3390/ma17030562 (PMC10856753; doi:10.3390/ma17030562)
Supplement: Supplementary file 1 [file materials-17-00562-s001.zip › materials-2807484-supplementary.pdf]

materials

Supporting Information

Mn(II)-Activated Zero-Dimensional Zinc(II)-Based Metal Halide Hybrids with Near-Unity Photoluminescence Quantum Yield

Chengyu Peng [1], Jiazheng Wei [1], Lian Duan [1], Ye Tian [2,*] and Qilin Wei [3,*]

[1] Traffic Information Engineering Institute, Guangxi Transport Vocational and Technical College, Nanning 530004, China; pengchengyu@gxjtc.edu.cn (C.P.)
[2] School of Semiconductors and Physics, North University of China, Taiyuan 030051, China
[3] School of Chemistry and Chemical Engineering, Shandong University, Jinan 250100, China
* Correspondence: tianye080t@163.com (Y.T.); qlwei@sdu.edu.cn (Q.W.)

Experimental method and characterization

Chemicals

Trans-2,5-Dimethylpiperazine ($C_6H_{14}N_2$, 98.0%), zinc bromide ($ZnBr_2$, AR, 99%), manganese (II) bromide tetrahydrate ($MnBr_2 \cdot 4H_2O$, 98%) and hydrogen bromide (HBr, ACS, 48 wt% in water) were all purchased from Macklin. Ethanol (EtOH; Guangdong Guanghua Sci-Tech Co., Ltd, AR) was used without any purification.

Synthesis of Mn^{2+}-doped (TDMP)$ZnBr_4$ crystals with Mn^{2+} feed ratio = 0, 10%, 20%, 40%, 60%

Mn^{2+}-doped (TDMP)$ZnBr_4$ crystals were synthesized using a solvent thermal method. Equal proportions of $C_6H_{14}N_2$ and $ZnBr_2$ were added to a 25 mL stainless steel autoclave with 3mL HBr. The feeding ratio of Mn^{2+} was calculated based on 0%, 10%, 20%, 40% and 60% of Cd^{2+} or Zn^{2+} content. The mixture solution was heated at 160 °C for 4 h and then cooled slowly to room temperature. The precipitated Mn^{2+}-doped (TDMP)$ZnBr_4$ was washed with ethanol and dried at 80 °C for 8 h.

Material characterization

A SMARTLAB 3KW X-ray diffractometer with Cu Kα radiation (λ= 1.54059 Å) was used to collect powder X-ray diffraction data in the 2θ range of 10–60°. Scanning electron microscopy (SEM, Hitachi SU8020) was used to observe the morphology. Energy-dispersive spectrometry (EDS, Oxford X-Max Aztec) was used to determine the element composition and distribution. The Raman spectrum and excitation power-dependent PL spectra were characterized using a WITec alpha300R Raman fluorescence spectrometer with a 633 nm laser as an excitation source. The photoluminescence (PL), PL excitation (PLE) spectrum, time-resolved photoluminescence (TRPL), photoluminescence quantum yields (PLQYs), temperature-dependent PL spectra and temperature-dependent PL lifetime were obtained using a Horiba Jobin Yvon Fluorolog-3 spectrometer. A Lambda 750 ultraviolet–visible spectrophotometer was used to measure the absorption spectrum.

Computational Details

The projector-augmented wave method was used to calculate the band structure, as implemented in the Vienna Ab initio simulation package (VASP). The generalized gradient approximation of the Perdew–Burke–Ernzerhof parameterization was used for the exchange and correlation functional. The kinetic energy cutoff of 500 eV and a 4 × 4 × 4

Monkhorst–Pack k-mesh for the wavefunction basis set were used. The energy convergence criterion was set as 1.0 × 10^{-6} eV for structure relaxations.

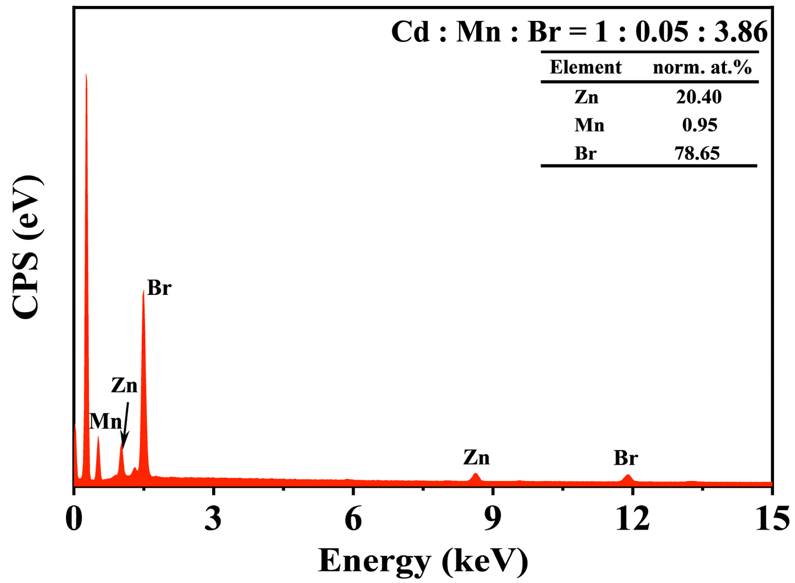

Figure S1. The EDS spectra of (TDMP)$ZnBr_4$ with 20%Mn^{2+} ion feed ratio.

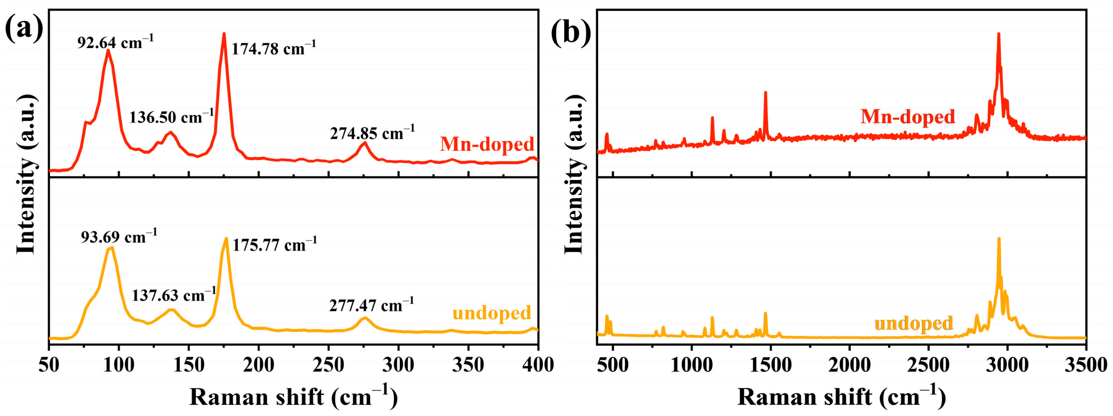

Figure S2. The inorganic part (**a**) and organic part (**b**) of Raman spectra of pure (TDMP)$ZnBr_4$ and Mn^{2+}:(TDMP)$ZnBr_4$.

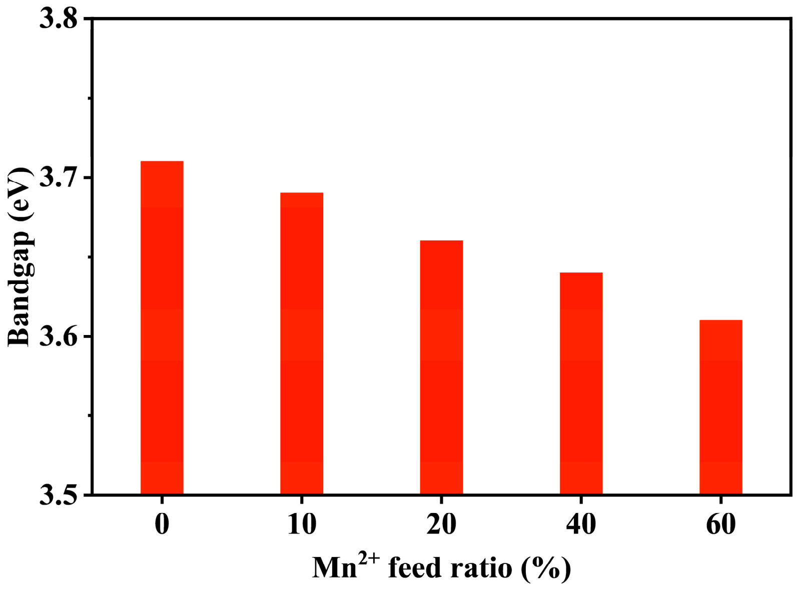

Figure S3. The bandgap value change trend of (TDMP)$ZnBr_4$ under different Mn^{2+} ion feed ratios.

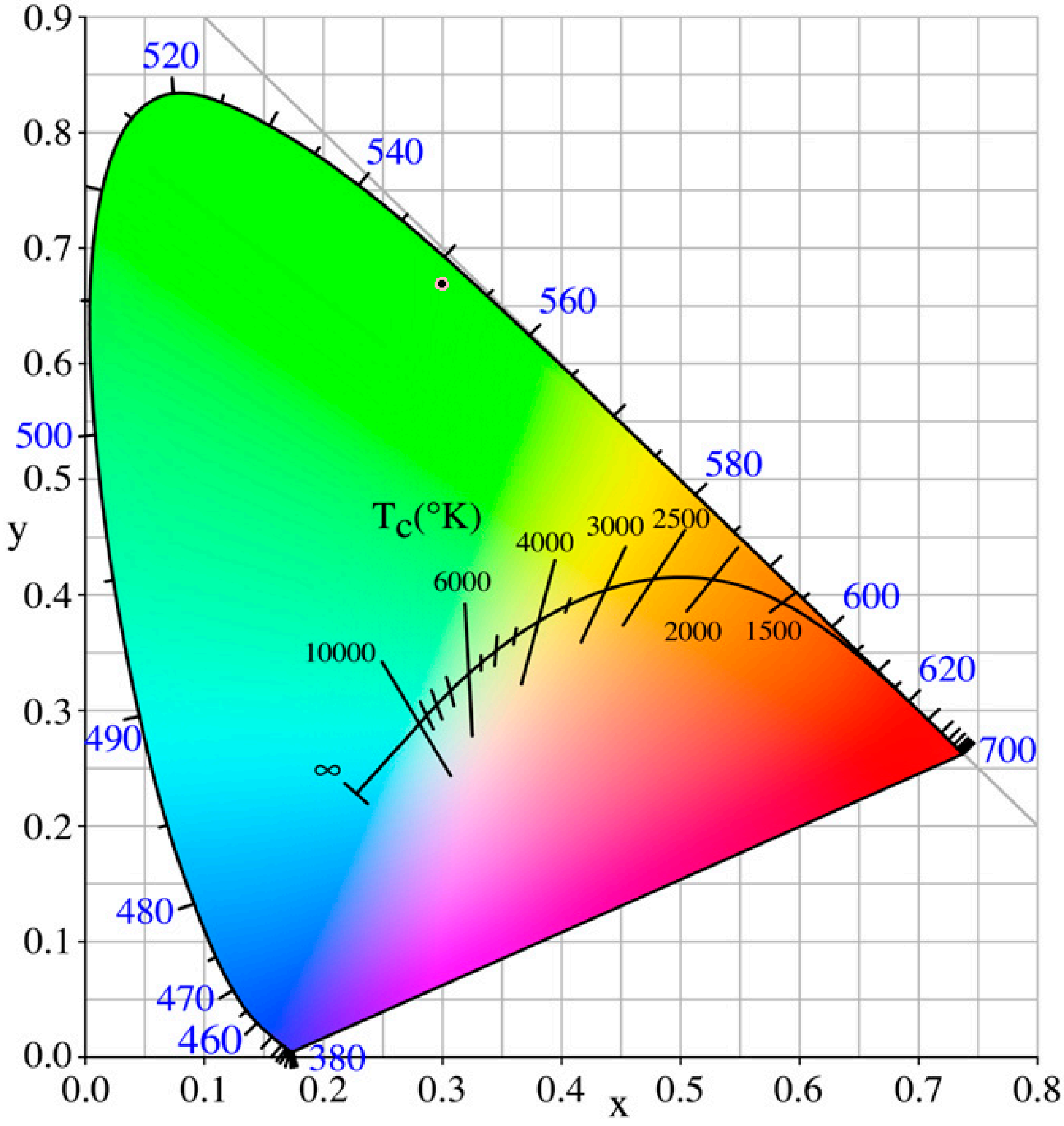

Figure S4. The CIE coordinate of Mn^{2+}:(TDMP)$ZnBr_4$.

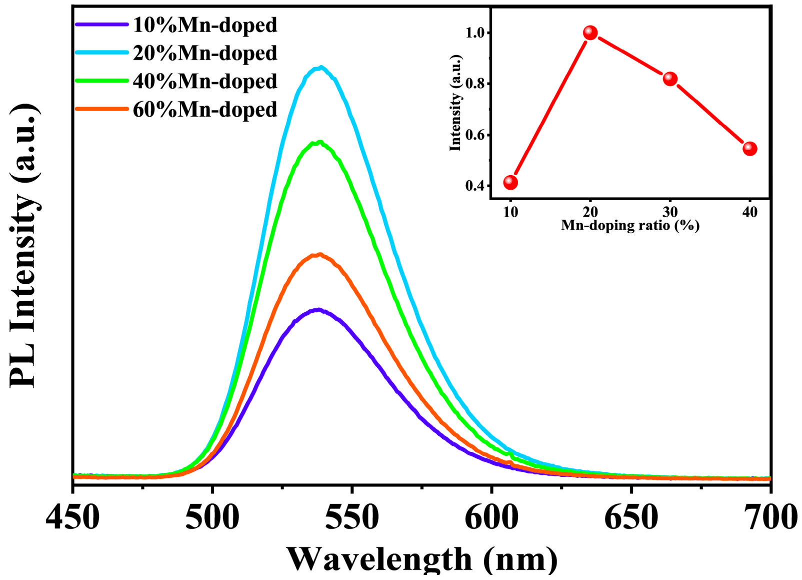

Figure S5. The PL spectra of (TDMP)$ZnBr_4$ under different Mn^{2+} ion feed ratios. Insert: the PL intensity change trend.

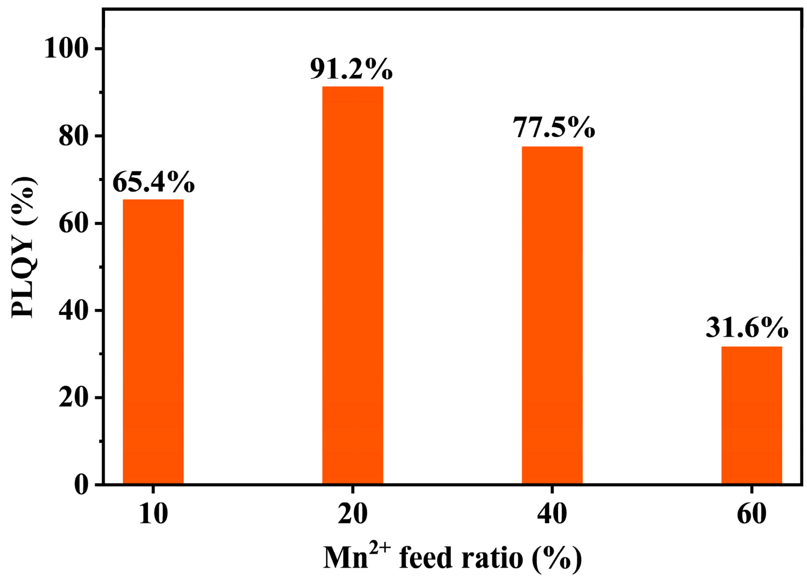

Figure S6. The PLQY of (TDMP)$ZnBr_4$ under different Mn^{2+} ion feed ratios.

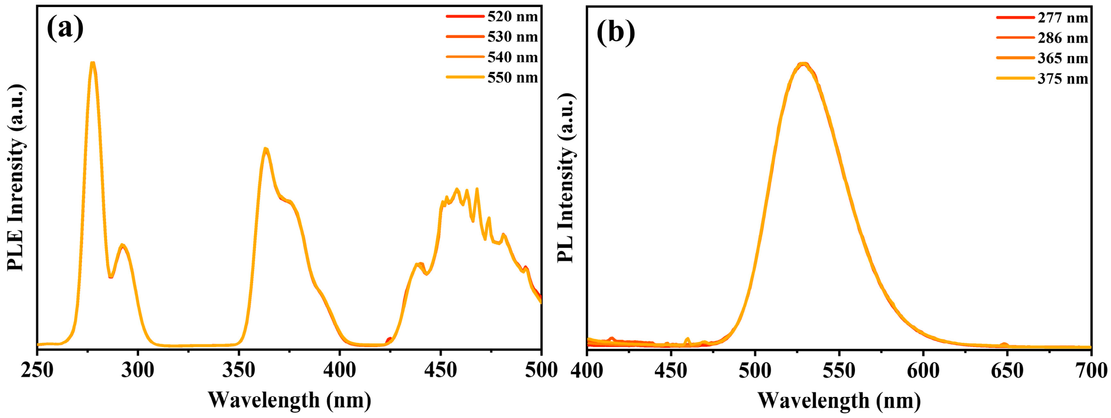

Figure S7. (**a**) The PLE spectra of Mn^{2+}:(TDMP)$ZnBr_4$ under different emission wavelengths. (**b**) The PL spectra of Mn^{2+}:(TDMP)$ZnBr_4$ under different excitation wavelengths.
